# A Scoping Review of On-Farm Colostrum Management Practices for Optimal Transfer of Immunity in Dairy Calves

**DOI:** 10.3389/fvets.2021.668639

**Published:** 2021-07-19

**Authors:** Lisa Robbers, Ruurd Jorritsma, Mirjam Nielen, Ad Koets

**Affiliations:** ^1^Population Health Sciences, Faculty of Veterinary Medicine, Utrecht University, Utrecht, Netherlands; ^2^Wageningen Bioveterinary Research, Lelystad, Netherlands

**Keywords:** colostrum management, calf feeding, immunity, colostrum storage, milking, scoping review

## Abstract

Newborn calves are agammaglobulinemic and rely for their first immune protection almost completely on the transfer of immune constituents via colostrum. Inadequate colostrum management practices such as on-farm colostrum storage practices and colostrum feeding methods could affect immune components in colostrum and subsequently immune status of the newborn calf. We conducted a scoping review to identify all literature on the interactions between several colostrum management factors and immunological colostrum quality and passive transfer of immunity. Three major stages were defined: milking methods, colostrum treatment and storage, and administration procedures. Separate CAB Abstracts searches were performed for each of the subjects of interest. The search process was completed on November 9, 2020. Colostrum should be milked as soon as possible, as IgG concentration diminishes over time, probably due to dilution. To minimize bacterial contamination, it is advised to pasteurize colostrum in small batches at maximal 60°C for 30 or 60 min. Freeze/thawing of colostrum does not or only slightly affect IgG concentrations, as long as thawing is done au bain-marie and temperature does not exceed 40°C. In on-farm situations, it is difficult to determine the volume that should be fed as the variables contributing to the absorption of IgG by the newborn calf are many and include the quality of the colostrum, the bacterial contamination, the time interval between birth and first moment of feeding and the weight of the calf. Despite all knowledge regarding optimal colostrum management strategies, it remains challenging to predict the effects of certain colostrum management choices in field conditions. Therefore, we recommend measuring the colostral quality, weighing the newborn calf, adjusting the feeding volume accordingly to ensure optimal colostrum intake for each calf.

## Introduction

The first weeks of life are a critical period in the development of the newborn calf as it is very susceptible to various pathogens. Cows, just like other ruminants, have an epitheliochorial placenta which prevents the transfer of passive immunity to the neonate during gestation. Hence, newborn calves are agammaglobulinemic and rely for their first immune protection almost entirely on the transfer of immune constituents through the first ingested colostrum. Immunoglobulin (Ig) G is traditionally considered critical in assessing colostrum quality because it is the most abundant Ig (IgG accounts for ~85–90% of the total Ig in colostrum) (1). Two major subtypes of IgG can be distinguished in colostrum: IgG_1_ which makes up 80–90% of the total IgG, and IgG_2_. Besides IgG, also IgM (7%) and IgA (5%) are present in colostrum (1). Where IgG appears in monomeric form, IgA and IgM appear in multimeric form. The neonate absorbs all three immunoglobulin classes, but IgA is partly released back into the intestinal lumen to for local, mucosal protection (2). IgM is mainly involved in primary immune response. Because IgA and IgM have a relatively short half-life (3–4 days) compared to IgG (21–28 days) (3, 4), they only perform protective functions in the neonate for a short time. Usually, colostrum quality is expressed in terms of IgG concentration, which is highly variable between cows (5, 6). Colostrum of high quality typically contains a concentration of >50 g/L, while lower concentrations indicate colostrum of low quality (1). It has been widely accepted that a calf should have a serum IgG concentration of at least 10 mg/mL between 24 and 48 h after birth (1). By definition, there is a failed transfer of passive immunity (FPI) when this criterium is not met, which is correlated with higher incidence of early calf morbidity and mortality as reviewed extensively by many (1, 7, 8). More recently, Lombard et al. suggested to revise this guideline into a new standard, in which the dose-dependent relationship between calf serum IgG concentration and calf morbidity is taken into account (9). In practice, achieving successful transfer of passive immunity through colostrum is one of the main challenges in calf rearing.

Optimal on-farm colostrum management is essential to ensure adequate transfer of passive immunity and provide the best start for newborn calves. Many studies have been conducted to define optimal management strategies for colostrum feeding. Farmers, veterinarians and feed advisors have adopted the “Three Q's” as a general guideline for providing colostrum: Quantity, Quality and Quickness of feeding. In addition, sometimes two “Q's” are added: “Quantifying the transfer of immunoglobulins” and “sQueaky clean” (10). In practice, farmers strive to provide a sufficient amount of high quality colostrum as quickly as possible to ensure adequate transfer of passive immunity. Meanwhile, bacterial contamination should be minimized and to ensure proper absorption by the calf, serum IgG concentrations should be monitored between 24 and 48 h of age. An overview of all existing knowledge on colostrum management and its effects on colostrum quality and immune transfer to the calf is lacking. We therefore conducted a scoping review on the effect of several on-farm management factors on immunological colostrum quality and transfer of passive immunity by the calf. We grouped the effects into (1) the milking methods, (2) colostrum treatment and storage and (3) administration procedures. The first two are aimed to optimize the quality of colostrum with regards to IgG concentration, while the latter aims to achieve most efficient colostral IgG uptake by the calf. Our research question for this scoping review was defined as: What is the up-to-date evidence on the interactions between on-farm colostrum management, colostrum quality and passive immunity.

## Materials and Methods

### Literature Search

Separate CAB Abstracts searches were performed for each of the subjects of interest. All searches included the following terms: (cow OR cattle AND colostrum).mp. The “mp” includes all articles with selected key terms in their abstract, title, original title, broad terms, heading words, identifiers and cabicodes. To identify papers concerning milking methods, these general terms were combined with the term (milking).mp. To identify papers describing storage and treatment methods, the general terms were combined with the terms (storage OR treatment OR heating OR pasteurization OR pasteurization OR temperature OR refrigeration OR thawing OR heat OR frozen).mp. Finally, papers describing feeding methods were identified by extending the general terms to (cow OR cattle OR calves OR calf AND colostrum).mp, and combining these with (bucket feeding OR tube feeding OR esophageal feeding OR feeding method OR feeding technique OR feeding frequency OR bottle feeding OR suckling OR calf feeding).mp.

#### Inclusion Criteria

This review only includes peer-reviewed articles presenting primary research to farm-related management of fresh bovine colostrum for calf feeding and the effects on (colostral and/or serum) immunoglobulins, leukocytes and other potential immune components. Articles were written in English or Dutch, and articles were included only if full text was available. Articles published up until November 9, 2020 were included. Additional selection criteria were included for the separate topics. All selected papers contain studies with common breeds of dairy cattle that were healthy and did not undergo specific treatment before the study period (e.g., no specific vaccination prior to calving or dry feeding strategies). For the selection of papers on milking strategies, only papers addressing milking of dairy cows were included. For selecting papers describing storage and treatments, reports involving bacterial counts were included during the first selection phase. During the second evaluation (full text articles), studies on bacterial counts were only included if they contained information on immune parameters and did not involve *in vitro* studies on the effects of specific bacterial strains. For selecting the papers evaluating feeding methods, papers were included when colostrum feedings were from fresh bovine colostrum, when feedings occurred within 24 h after birth, and when serum immune concentrations were assessed at least within 24–48 h after birth. For suckling of the calf, beef cattle studies were selected as well. The first author performed data retrieval. For each search, titles and abstracts were scanned for the selection criteria described above. Of the remaining papers, the main text was evaluated for relevance. In addition, the first author assessed the articles when uncertainty existed on whether or not to include a paper; this was discussed with the second author.

## Results

### Milking Methods

#### Search Methods

**Figure 1:**
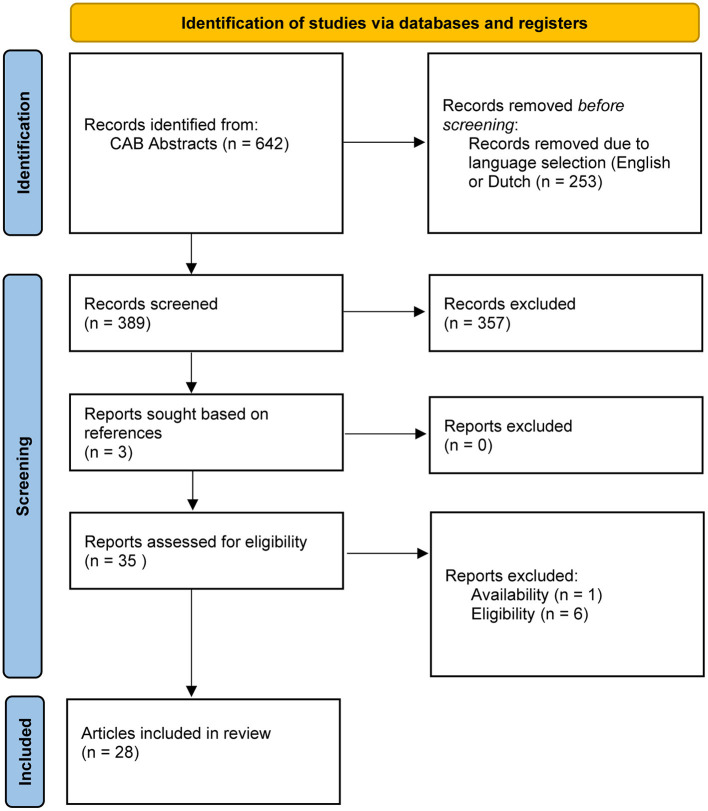
Flowchart depicting the article selection process for the subsection “milking methods.”

There were 642 records identified using keywords for “milking methods” as described. Two hundred fifty-three records were excluded because they were not in English or Dutch. Title and abstract first screening led to the exclusion of 357 papers that did not contain original research data (such as reviews), were not peer reviewed (such as conference proceedings), and/or did not fit the inclusion criteria. The remaining 32 articles were assessed full text. Three reports were added based on references in full-text read articles. One paper was excluded for it was unavailable full text; six others were excluded because they did not fit the selection criteria. A flowchart summarizing the selection process can be found in Figure 1. In total, 28 articles were included, of which 2 animal studies and 26 population studies. An overview of the study types can be found in Table 1.

**Table 1:** Overview of the study types included in the results, separate columns for each of the sections: colostrum milking, treatments and storage, and administration procedures.

	**Milking methods**	**Colostrum treatments and storage**	**Administration procedures**	**Total**
Animal studies	2	24	36	62
Laboratory studies		26		26
Population studies	26	6	18	50
Total	28	56	54	138

*Note that the number of total study types for “Colostrum treatments and storage,” 56, does not match the number of articles included in our review. This is because some articles present more than one study type, e.g., a combination of laboratory study type and animal study type*.

### Time Between Calving and First Colostrum Milking

Timing of first colostrum milking is thought to affect immunoglobulin concentrations of colostrum. Many studies agree that by shortening the interval between parturition and milking, the concentration of Ig in colostrum is higher, consequently improving colostrum quality (6, 11–20). Still, the time after which the immunoglobulin concentration starts to decline is debated. Conneely et al. found that colostral IgG concentrations in milkings occurring 0–3, 3–6, or 6–9 h post-partum were similar and reports a significant reduction over 9h post-partum (13). These results are confirmed by other studies, that found no negative correlation between colostral IgG and time interval when milked within 3–9 h post-partum (21–24). Kessler et al. (6) reported however that milking colostrum within 3 h post-partum results in significantly higher IgG concentration compared to milking colostrum later. Between 3 and 12 h post-partum the concentration remains relatively similar (6). Both Kruse and Moore et al. found highest colostral immunoglobulins in colostrum milked within 2 h (11, 12). Morin et al. and Conneely et al. described, respectively, a 3.7 and 1.1% decrease in colostral IgG concentration with every hour the milking was delayed (13, 14), suggesting milking directly after calving is most optimal. A delay in first colostrum milking may result in dilution of the immunoglobulin content, as colostrogenesis and thereby the transfer of immunoglobulins into colostrum is believed to end at parturition. As the time interval until first milking increases, lactogenesis is initiated and milk yield increases (13). Several studies found a negative correlation between first milking yield and immunoglobulin concentration (13, 18, 20, 22, 25, 26), indicating that delayed milking leads to dilution of the colostrum, thus reducing the antibody concentration. However, Conneely et al. (13) argue that even after adjusting for the colostral weight, cows that were milked at a later time still produced colostrum with lower IgG concentration. Additionally, some studies did not find an association between increased first colostrum yield and the concentration of immunoglobulins (12, 23, 24, 27, 28). Kessler et al. (24) propose that the process of colostrogenesis does not abruptly end at parturition and that immunoglobulin transfer continues a few hours after calving. This would explain their findings that colostrum milked within 30 min post-partum contains lower IgG concentrations than colostrum milked 3 h after calving (24).

### Fractional Milking Strategies

Following parturition, colostrum can be milked out completely, but it is also possible to e.g., collect only the quantity needed to cover the needs of the calf and milk the cow partially. Using different fractions of colostrum (other than milking completely) may affect the colostral antibody concentrations. Stott et al. (29) studied whether colostrum collected from the cisterns contained higher concentrations of immunoglobulins. From each quarter, 100 ml was collected, after which the remaining colostrum was milked completely. For IgG, IgM and IgA no concentration differences were observed between the cisternal and the complete colostrum (29). Other studies found similar results (30–32). However, Ontsouka et al. obtained samples on day 2 after calving, which usually contains lower levels of immunoglobulins (31). Godden and Hazel (33) assessed IgG concentrations in different fractions of colostrum. During the entire process of colostrum collection, every 30 s a 10 mL sample of colostrum was collected into a syringe through a sampling port that was located in the milk line. This enabled them to make a clear distinction of the separate fractions. In contrast to other findings, they describe a clear difference in IgG concentration between cisternal and composite samples. Moreover, they found higher IgG concentrations in cisternal colostrum compared to the first quartile, the first half and the first three quarters of the first milking. Milking 25, 50, and 75% of colostrum showed no IgG concentration differences (33). Despite contradicting observations regarding fractional milking, the use of cisternal fractions of the first streaks of colostrum to estimate its quality is discouraged, as those samples may not represent total colostrum quality (33). Sroka et al. suggest milking completely and save the remaining colostrum after first feeding for the next feedings, since consecutive milkings from cows milked out completely contain lower IgG concentrations (34).

The effect of milking individual quarters at different time points as fractional milking strategy is not extensively studied. Madsen et al. (35) reported some small differences in IgG concentrations when one quarter was omitted for milking, but omitting one quarter did not affect total IgG yield. This was explained by the neutralization of all quarters in the fourth milking (35). Also Gomes et al. and Kessler et al. did not find any significant differences in IgG, IgM or IgA concentration between quarters (24, 36), although large variations in production between quarters were observed (24). Baumrucker et al. on the other hand found that the front quarters produced colostrum with a higher concentration of IgG compared to the rear quarters (37).

### Colostrum Treatment and Storage

#### Search Methods

**Figure 2:**
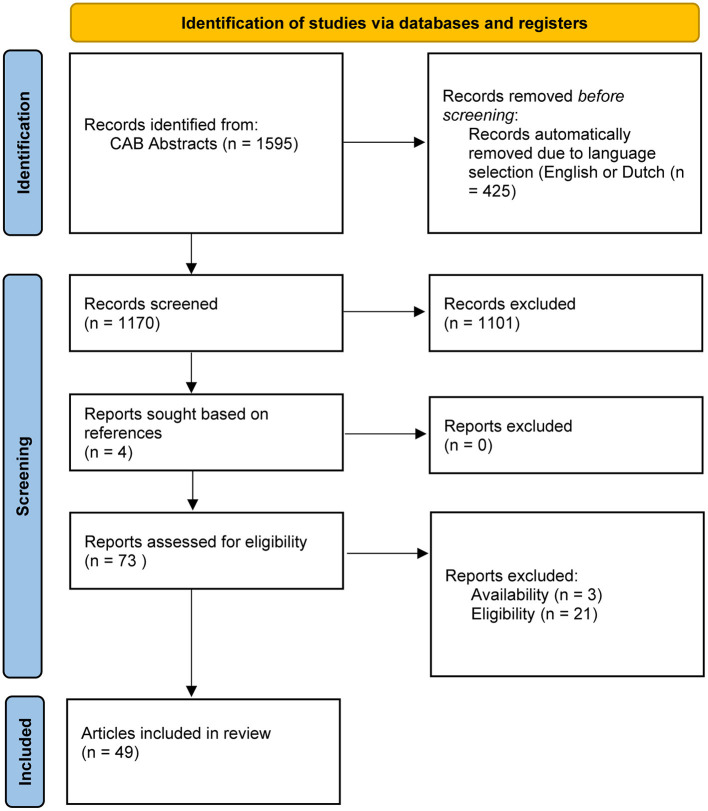
Flowchart depicting the article selection process for the subsection “colostrum treatment and storage.”

There were 1595 records identified using keywords for “milking methods” as described. Four hundred twenty-five records were excluded because they were not in English or Dutch. Title and abstract first screening led to the exclusion of 1101 papers that did not contain original research data (such as reviews), were not peer reviewed (such as conference proceedings), and/or did not fit the inclusion criteria. The remaining 69 articles were assessed full text. Four reports were added based on references in full-text read articles. Three papers were excluded for they were unavailable full text; twenty-one others were excluded because they did not fit the selection criteria. A flowchart summarizing the selection process can be found in Figure 2. In total, 49 articles were included, of which 24 animal studies, 26 laboratory studies and six population studies. An overview of the study types can be found in Table 1. The numbers in Table 1 for “Colostrum treatment and storage” add up to 56 instead of 49. This is because seven articles combined two study types, for example laboratory study and animal study.

To review the effects of storage, we decided to make a distinction between short term and long term storage or treatment effects. We defined short term storage or treatment as on-farm practices applied to freshly milked colostrum used to feed calves within 48 h after milking. In case dams cannot produce a sufficient volume or high colostrum quality, there is a need for stored colostrum from other cows that calved earlier. Thus, it is important to evaluate the effect of long term storage of colostrum on quality as well. We defined long term storage a method applied to store or treat fresh colostrum for a period longer than 48 h after milking.

#### Short Term Storage

Some studies have investigated the effect of short term storing conditions on the presence of (pathogenic) microorganisms in colostrum. Fewer studies looked into effects on immunological parameters.

##### Storing at Different Temperatures

Storing colostrum at room temperature or at 4°C did not affect IgG concentration (38). However, an association was found between increased bacterial counts (>1,000,000) and decreased calf serum IgG levels after storage at higher temperature (22°C) (39). With respect to storing colostrum in a refrigerator, Langel et al. note that refrigeration (4°C) up to 8 h did not affect cell viability (40), whereas effects of refrigeration for a longer period are yet unclear. We did not find other literature investigating effects of short term storage of colostrum such as temperature and/or duration, on colostral Igs or other immune components in colostrum.

##### Pasteurizing Colostrum

Pasteurization of milk is widely used to eliminate the number of microorganisms to a minimum at which milk can safely be used for human consumption. Pasteurization can also be applied to minimize the amount of pathogens in colostrum in order to minimize health risks for the calf. As the effects of pasteurization on pathogen elimination are widely ascertained, we focus on the effects of pasteurization on the immunological content of colostrum by looking at immunoglobulins in particular. In addition, we payed attention to the apparent efficiency of absorption (AEA) of immunoglobulins by the neonate.

*Effects on Colostrum IgG.* Protein structure can be altered by temperature and therefore heating of colostrum can influence availability and functionality of proteins including immunoglobulins. The majority of studies investigating pasteurization effects focus on colostral or calf serum IgG. In all existing literature a variety of combinations regarding temperature and duration of pasteurization is applied and therefore these studies are a challenge to compare. We summarized the results of the existing literature in Supplementary Table 1. The majority of studies show that colostral IgG concentration is not (41–53) or only slightly affected (54–57) by heating at <60°C for either 30 or 60 min. In contrast, heating of colostrum above 60°C frequently resulted in significant loss of colostral IgG (41–48, 50, 54, 58–60). Heating colostrum at a temperature of 60°C for 30 or 60 min leads to significant reduction in bacterial counts, while viscosity remains similar and there is only a slight reduction in colostral IgG concentration (54). Several studies noted that the loss of IgG depended on the original quality of colostrum and found that colostrum of high quality suffered from a greater reduction in IgG concentration compared to colostrum of low quality (41, 47, 58). Meylan et al. argue that high quality colostrum is more likely to aggregate, leading to protein clump formation, which results in denaturation of the protein (58). However, different results were obtained by Balthazar et al., who observed a greater IgG losses for low-quality colostrum compared to high-quality colostrum (61).

*Effects on Serum IgG and Apparent Efficiency of Absorption.* While pasteurization of colostrum can result in slight reduction of colostral IgG concentration, it has been suggested that calf serum IgG levels are potentially increased when colostrum is pasteurized. The study by Johnson et al. (42) was one of the first to describe that there was no noteworthy difference in IgG observed between pasteurized and unpasteurized colostrum, but calf serum IgG levels were significantly higher in calves fed pasteurized colostrum (22.34 vs. 18.07 mg/mL) (42). Subsequent studies revealed similar results: heat treatment of colostrum at 60°C for 30 or 60 min preserved both viscosity and IgG levels of colostrum, while serum IgG and AEA was higher in calves fed the heat treated colostrum compared to calves fed unheated colostrum (43, 44, 47–49, 57, 62, 63). Possibly, degradation and denaturation of proteins that would otherwise compete with the intestinal absorption of IgG contributes to increased IgG absorption after pasteurization. Also, immunoglobulins transferred with colostrum could bind to bacteria, thereby inhibiting them from passing the epithelial barrier. When the number of bacteria is reduced by pasteurization, this can lead to increased amounts of free immunoglobulins which can pass the epithelial barrier. This theory is supported by the work of Gelsinger (62). Colostrum with a high bacterial count—but with similar IgG concentration—resulted in decreased AEA compared to colostrum low in bacterial count (62). However, in an earlier study performed by Elizondo-Salazar no such difference in AEA was noted (44). Perhaps entire bacterial dead cells or their fragments may still attach to colostral immunoglobulins in the gut and thus reduce their absorption.

Although pasteurization of colostrum *above* 60°C is generally associated with decreased colostral immunoglobulins, several studies noted that this does not automatically lead to decreased calf serum concentrations. Tyler et al. (64) studied the effects of pasteurizing at different temperatures and found that heating colostrum to 63°C for 30 min did not affect serum IgG in calves compared to unpasteurized colostrum. Pasteurization at 76°C did lead to lower serum IgG levels (64). Also, Bush et al. (65) even found calf serum IgG to be increased 12 h after calves ingested pasteurized colostrum (63°C) compared to calves fed unpasteurized colostrum. In subsequent measurements, this difference diminished (65). Also Lakritz et al. found no significant effect on serum IgG concentrations between calves fed pasteurized (76°C) or unpasteurized colostrum (66). However this study was performed with a small sample size and results should be interpreted with care. Stabel reported that over the long term (1 month) no differences in calf serum IgG are found between calves fed pasteurized colostrum (65°C) and calves that suckled fresh unpasteurized colostrum (67).

While the majority of the studies focus on loss of colostral IgG concentration, few paid attention to loss of IgG functionality. McMartin et al. performed serum neutralization assay to determine colostral antibody activity in samples pasteurized at either 60 or 63°C. While no adverse effects of pasteurization on antibody activity were found, the authors emphasize that these results should be interpreted with care, as many samples were lost due to congealing in the process of pasteurization (41).

*Effects on Other Immunological Factors.* Lactoferrin is an immune component of colostrum, which is known to possess immunomodulatory and antimicrobial properties and is therefore interesting to look into. A few studies have looked into the effects of heating colostrum on lactoferrin levels. Pasteurization at 60, 63, 72, and 76°C lead to significant reduction of lactoferrin concentrations in colostrum and in calf serum, as visualized in Supplementary Table 2 (66, 68, 69). These results largely agree with the study by Shimo et al., who recorded significant aggregation and denaturation of lactoferrin at temperatures above 63°C, especially when pH was increased as well. Below 63°C, no significant denaturation occurred (70). Other immunological compounds in colostrum such as cytokines were studied as well. Gelsinger and Heinrichs (55) found that neonatal absorption of colostral interferon-γ was not affected by pasteurization at 60°C for 60 min. On the other hand, interleukin 1β concentration in calves receiving heat-treated colostrum was decreased. The authors suggest that the neonatal immune response is not inhibited by heat treatment of colostrum (55). In the same study, calves were subcutaneously challenged with ovalbumin to determine B cell activity and thus antibody production. Calves receiving heat treated colostrum showed higher ovalbumin specific IgG levels to this challenge indicating that neonatal B-cell function was not affected by heat treatment of colostrum. However, calves receiving unheated colostrum tended to recover their average daily gain faster after the immune challenge. During the Minnesota Dairy Health Conference in 2012 Godden et al. discussed their study investigating whether viability of colostral immune cells was affected by the process of pasteurization. Indeed, viability was reduced but not completely eliminated after pasteurizing for 60 min at 60°C. Also the authors state that although viability of colostral immune cells is reduced, more research is required regarding functionality and biological meaning of colostral cells (45).

#### Long Term Storage and Treatments

##### Freezing and Thawing of Colostrum

As freezing will always be inextricably associated with thawing, investigating the effect of freezing on colostrum quality should be combined with thawing, just as thawing should be studied in combination with freezing. Hence, we will review studies investigating freezing and/or thawing and combine results in our analysis.

*Effects on Colostrum IgG*. Several studies examined the effects of freeze/thawing on IgG content of colostrum and serum IgG (Supplementary Table 3). To our best knowledge, there are no studies investigating thawing at room temperature.

Wiking and Pedersen (71) studied several ways to thaw and heat colostrum in a microwave oven. Heating of refrigerated colostrum samples did not directly lead to a loss of IgG. Thawing of frozen colostrum by microwave resulted in unevenly heated colostrum and clotting, however no information of IgG concentration was reported (71). Jones et al. (72) examined thawing by microwaving at two different microwave settings (325 and 650 Watt) and found no differences in IgG or IgM content as compared to thawing au bain-marie (45°C). However, small losses of IgA were found (72). Balthazar et al. found that increasing the power of a microwave was associated with a significant greater loss of IgG_1_: 20% loss at 200 W vs. 31% loss at 350 W (61). Heating to 50 and 60°C au bain-marie resulted in a similar IgG loss as heating at 40°C (8%), while heating above 60°C resulted in a significant (26%) reduction in IgG1. This is in line with other studies on pasteurization showing greater loss of IgG when colostrum is heated above 60°C. Losses of IgG_1_ were greater for the low quality and thus low IgG_1_ fresh samples compared to the high quality fresh samples (61). With regard to repeated freeze/thawing, Haines et al. did not find significant changes in IgA, IgM or IgG concentrations in a single colostrum sample after multiple freeze-thawing cycles with a water bath at 37°C (73). However, since this study only included one sample these results should be interpreted with care. A larger study by Morrill et al. (74) showed that freeze/thawing for a single time does not reduce colostral IgG concentration. Compared to fresh colostrum, repeated freeze/thawing leads to a significant decrease of 7.8 and 7.7% for two and three freeze/thaw cycles, respectively (74). To our knowledge, no studies have examined how repeated freeze/thawing affects colostral immunoglobulin stability or function.

*Effects on Serum IgG*. Few studies looked into the effects of feeding colostrum after freezing/thawing on serum immunoglobulin concentration of neonate calves. Olson (75) found that both serum IgG_1_ and IgG_2_ levels were lower in calves fed au bain-marie heated (41°C) colostrum compared to calves fed microwave heated colostrum (312 W, heated to 41°C), while colostral IgG_1_, IgA and IgM concentrations were similar and the IgG_2_ was only marginally lower. The authors note that these results should be interpreted with care, since the size of the study was quite small (75). Holloway et al. and Donovan et al. found no significant differences in serum IgG concentration between calves fed frozen and thawed colostrum and calves fed fresh colostrum (76, 77).

*Effects on Other Immunological Factors*. With respect to other immune components of colostrum, Holloway et al. (78) studied whether freeze/thawing affected lactoferrin concentrations in colostrum and calf serum. No difference in lactoferrin concentrations was observed between fresh and frozen colostrum. Serum lactoferrin concentration on day 2 did not differ between the calves fed fresh or freeze/thawed colostrum, however on days 4 and 7 serum concentrations were higher in the freeze/thawed colostrum fed calves. Unfortunately, the authors were unable to determine whether the measured serum lactoferrin was derived from colostrum or from endogenous origin (78). In addition to immunoglobulins and lactoferrin, colostrum contains maternal leukocytes as well. The general opinion is that leukocytes remain viable and functional under certain specific circumstances: the optimal temperature for mammalian cells is 37°C, increasing the temperature above 42°C leads to denaturation of proteins and destruction of the cell, and freezing leads to intracellular ice crystal formation and thereby to damage and even lysis of cells. The assumption that colostral cells are indeed destroyed during freezing is supported by the studies by Novo et al. (79, 80). Using Trypan blue to check for cell viability, they report that no viable cells were found in their freeze/thawed colostrum (79, 80). Many papers studying the effects of leukocytes in colostrum make use of freeze/thawing for their control group of cell-free colostrum. Donovan et al. (77) studied the functionality of maternal colostral cells in neonatal calves. Calves were fed fresh colostrum, freeze/thawed colostrum or cell-free colostrum, all from dams vaccinated against Bovine Viral Diarrhea Virus (BVDV). The group fed fresh colostrum, with colostral cells, showed increased *in vitro* proliferative responses after stimulation with BVDV. Both the thawed and cell-free colostrum groups did not. The observed difference was attributed to the functionality of transferred maternal cells (77). These results combined with other results of studies using freeze/thawing as a valid method to lyse maternal cells in colostrum, indicate that freezing of colostrum indeed destructs colostral leukocytes (40, 79–81). On the contrary, Stieler et al. (82) found increased neutrophilic activity in calves fed fresh frozen colostrum compared to calves fed fresh colostrum, although not significant. No differences in activation capacity was observed after follow up with milk replacer for 21 days. The authors attributed the increased neutrophilic activity in the frozen/thawed colostrum fed calves to the release of transfer factors by lymphocytes in response to freezing, leading to stimulation of the cellular immune response (82).

##### Other Treatments

Few studies have experimented with high pressure techniques to eliminate bacterial counts in colostrum. Masuda et al. (83) reported effective suppression of bacterial growth for 9 days at 4°C after treating colostrum at 300 and 400 MPa for 10 min. Up to 300 MPa, IgG remained intact, but application of 400 MPa resulted in altered viscosity of the colostrum and denaturation of IgG (83). Indyk et al. (84) and Foster et al. (85) found colostral IgG to be stable up to 400 MPa treatment, as long as duration was limited to 30 min. Increasing pressure (500 or 600 MPa) or duration resulted in increased denaturation and aggregation (85). Apparently, IgG in colostrum is more stable compared to IgG isolated from colostrum. Probably the colostral environment enhances the stability of immunoglobulins (84). The use of formaldehyde to preserve colostrum is sometimes applied in warm ambient climates when refrigeration is not an option. Mbuthia et al. found treatment with formaldehyde to be the best method for preserving immunoglobulin content in colostrum for up to 4 weeks compared to treatment with formic acid or natural fermentation (86).

### Administration Procedures

#### Search Methods

**Figure 3:**
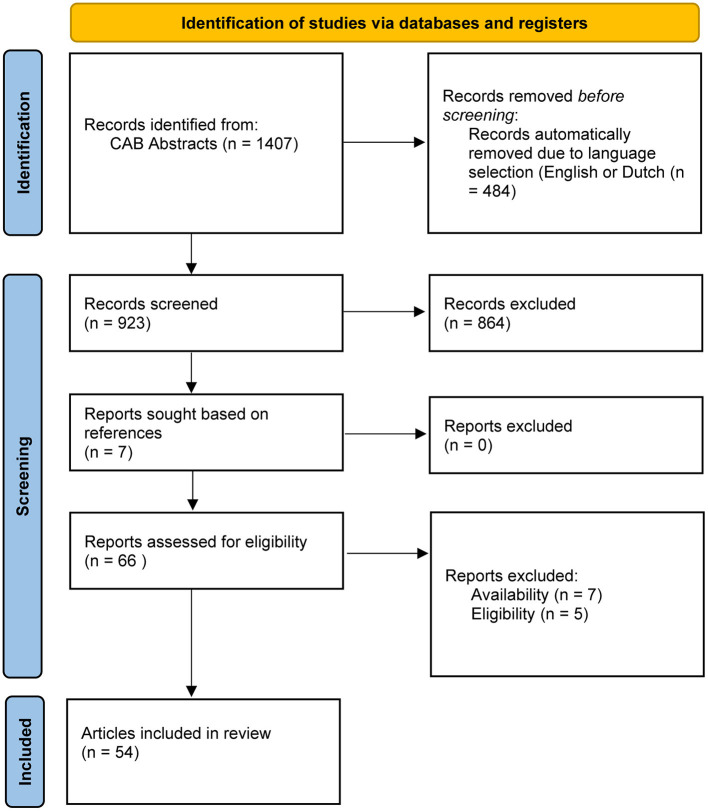
Flowchart depicting the article selection process for the subsection “administration procedures.”

There were 1407 records identified using keywords for “milking methods” as described. Four hundred eighty-four records were excluded because they were not in English or Dutch. Title and abstract first screening led to the exclusion of 923 papers that did not contain original research data (such as reviews), were not peer reviewed (such as conference proceedings), and/or did not fit the inclusion criteria. The remaining 59 articles were assessed full text. Seven reports were added based on references in full-text read articles. Seven papers were excluded for they were unavailable full text; five others were excluded because they did not fit the selection criteria. A flowchart summarizing the selection process can be found in Figure 3. In total, 54 articles were included, of which 36 animal studies and 18 population studies. An overview of the study types can be found in Table 1.

Achieving adequate passive immunity depends on timely ingestion of a certain level of immunoglobulins and the absorption ability of the calf. In this paragraph we discuss studies looking into the effect of time interval between birth and first feeding, volume of first feeding, and equipment used for colostrum feeding on calf serum Ig status.

#### Timing of First and Subsequent Feedings

While some studies failed to find an association between timing of first feeding and serum IgG concentration (87, 88), the study by Keulen et al. showed that besides volume and quality of first colostrum, also the timing of first feeding partially explains variation in calf serum IgG (89). Indeed, most studies agree that the ability to absorb IgG declines with delaying first colostrum feeding. Still, much uncertainty exists about the exact time period to which the first feeding can safely be delayed without affecting the ability to absorb IgG. An early study by Smith et al. noted that calves receiving their first colostrum at 10–12 h after birth, showed a larger proportion of low serum immunoglobulin cases compared to calves receiving their first colostrum feeding within 8 h. The study did not look further into the association between timing of first colostrum feeding and serum IgG or absorption rate (90). While some studies find it safe to feed the first colostrum up until 8 h of age without affecting IgG absorption (91), some claim the absorption rate starts to decline earlier, around 6 h of age (92) or even after 4 h (93–95). These last studies are in line with the study by Chigerwe et al., where no effect of time (<4 h) on FPT was observed (96). Another study by Chigerwe et al. recommended feeding of colostrum within 2 h of birth (97). The study by Osaka et al. shows a gradual decline in AEA over time for the first 12 h. After 12 h, a more rapid decline is observed (98). Some studies used models to estimate effects of delayed colostrum intake on calf serum IgG. One model showed that calf serum IgG concentration decreased with 2 mg/ml with every 30 min delay in colostrum intake (99), another found a decrease of with 0.32 mg/ml with each hour the colostrum ingestion is delayed (63). With respect to other immune components, Zanker et al. showed that delayed feeding of colostrum up to 24 h post-partum does not affect hematological values such as the total number of leukocytes in the neonate (100).

Absorption of IgG can be affected by the timing of the first colostrum, but also by timing of subsequent feedings. In the study by Fallon et al. it was shown that an additional feeding within 12 h significantly increased the serum immunoglobulin content (101). Timing of first colostrum feeding also affects the effectiveness of subsequent colostrum feedings. Calves fed colostrum within the hour with subsequent feedings at intervals of 8 h showed higher serum IgG than calves fed their first colostrum at 16 or 24 h followed by the same subsequent feedings (91).

#### Volume of Feeding

Many studies have attempted to determine the volume that should be consumed by the newborn calf to obtain first passive immunity. While investigations were carried out carefully, they differ from each other in terms of colostrum quality and timing of the first feeding. Therefore, a quantitative comparison of these studies is hardly possible. Some studies suggest to determine colostrum volume by means of the bodyweight of the newborn calf (102, 103). Conneely et al. propose that calves should ingest a volume corresponding to 8.5% of their body weight (within 2 h of age) (102). Some studies however claim that calves need to ingest a fixed amount of IgG to achieve successful transfer of passive immunity (serum IgG concentration >10 mg/ml) within 24–48 h of age. Some propose to provide at least 100 grams of IgG in total (26, 104), others deem this insufficient and suggest to feed 150–200 gram of IgG or possibly even more (97, 105). Morin et al. (105) conducted a series of experiments in which they extensively studied several combinations of colostral quality, volume and timing of feeding on serum IgG in newborn calves. They found that feeding 4 L of high quality colostrum (60.1 mg/ml) within 3 h after birth, followed by another 2 L at 12 h after birth resulted in highest serum IgG at both 24 (31.1 mg/ml) and 48 h after birth (30.4 mg/ml). In total, these calves consumed >360 g IgG. Calves receiving 240 grams of IgG, divided over 2 feedings of 2 L showed serum IgG concentrations of >20 mg/ml at both 24 and 48 h after birth (105), indicating sufficient transfer of passive immunity. For low quality colostrum (32.9 mg/ml), they recommend to feed an additional 2 L at 6 h of age next to feeding 2 L at birth and 12 h of age (105). Different conclusions were drawn by Jaster (106). For low quality colostrum he observed higher serum IgG levels in calves fed 4 L at once compared to calves receiving the same amount equally divided over a feeding moment at birth and at 12 h. For calves fed high quality colostrum, higher serum IgG levels were observed at 24 and 48 h when they were fed 2 L at birth followed by another 2 L at 12 h compared to calves fed 4 L at once, suggesting a maximal absorption capacity for IgG (106). Kaske et al. found also that providing 4 L containing 213 grams of IgG resulted in highest serum IgG levels (25.2 mg/ml). Feeding 2 L containing 97.4 grams of IgG resulted nevertheless in serum concentrations of 14.1 mg/ml, which is still above the threshold of 10 mg/ml (107). Hopkins and Quigley point out that providing 3.8 L in either one or two feedings results in equal serum IgG concentrations, considered colostrum is of good quality (>50 g/L IgG) (108). Chigerwe et al. (96) suggest calves should voluntary ingest as much colostrum as possible within 4 h of birth. Depending on the volume that is voluntarily ingested, they recommend a second feeding at 12 h of age (96). An on-farm study by Halleran et al. found calves fed 5.6 L within 12 h of age had significant higher serum IgG concentrations than calves fed 4L (109). A large retrospective study identifying risk factors contributing to FPT by Renaud et al. showed that feeding >6 L within the first 24 h of life was associated with decreased risk of FPT (110).

#### Feeding Methods

Many studies evaluated the effectiveness of several feeding methods to achieve successful transfer of passive immunity. Many studies looking into suckling with the dam showed that voluntary suckling (111, 112) and assisted suckling (113–115) can result in sufficient serum immunoglobulin concentrations. However, most studies report that suckling with the dam leads to high risks of FPI (26, 101, 116–120). A possible explanation is that there seems to be a large variation in the time it takes calves to voluntarily suckle (121) and this can increase up to more than 6 h (122). Studies by McBeath and Logan and Rajala and Castren therefore point out the importance of supervision when calves are left with the dam to suckle (99, 123). Additional studies showed that calves assisted to suckle after 6 h were still able to achieve proper transfer of IgG, while those not assisted showed FPI (95, 122, 124). Altogether, it appears that (assisted suckling) can lead to adequate transfer of passive immunity, but these methods do in general result in lower serum IgG levels when compared to active methods to deliver colostrum, such as esophageal or bottle feeding.

The use of a nipple bottle or (nipple) bucket is a common method for on-farm administration of colostrum. With respect to feeding with a nipple bottle, voluntary intake of colostrum by nipple bottle leads in many cases (31%) to insufficient (<2 L) ingestion of colostrum (125). While some studies reported higher immunoglobulin concentrations in calves that were nursed by the dam in comparison with calves fed by nipple bottle (112, 114), others found no difference (115, 126) or higher serum immunoglobulin concentrations for bottle fed calves (113, 127). When comparing feeding colostrum with a nipple bottle or bucket, no significant difference in serum IgG was observed at 24 or 48 h (128) or at 96 h (101). The use of an esophageal feeder is effective for achieving adequate passive immune transfer (129, 130). Several studies have compared the use of esophageal feeding of colostrum to other feeding methods (Supplementary Table 4). When compared to voluntary suckling, esophageal feeding seems to be the better option for preventing FPI (26). However, some studies in beef calves suggest that when calves are assisted for suckling, serum IgG levels are comparable to that of calves fed with esophageal feeder (131, 132). When bottle and esophageal feeding methods are compared, esophageal feeding results in either higher (107) or comparable serum IgG concentrations (133–138). Kaske et al. showed increased serum IgG levels in calves fed 4 L colostrum by esophageal tube compared to 2 L bottle fed calves (107), however these feedings differ both in volume and in method offered and therefore these results are difficult to compare. A more comprehensive study was carried out by Godden et al. (104) by investigating the differences between esophageal and bottle feeding at two different volumes. When using a fixed volume of 3 L colostrum replacer by either bottle or esophageal feeding, no differences in serum IgG were observed, which was later confirmed by Desjardins-Morrissette et al. as well (104, 136). However, feeding a smaller volume of 1.5 L resulted in higher serum IgG levels in bottle fed calves when compared to tube fed calves. This result implies a possible association between the method of feeding, the volume fed and the serum IgG in calves. As pointed out by Lateur-Rowet and Breukink (130) feeding colostrum with the use of an esophageal feeder led to failure of the esophageal groove reflex, consequently leading to colostrum being led to the rumen and reticulum, instead of the abomasum. Rapid flow from reticulorumen to abomasum was observed though (130). Kaske et al. estimated that the time of emptying the contents of the reticulum into the abomasum lies between 2 and 3 h (107). This may explain the results found by Godden et al. (104): when smaller volumes of colostrum are fed by drenching, the reticulum would not easily overflow and colostrum does not enter the abomasum quickly. However, when larger amounts are fed, the reticulum overflows and colostrum is directly transferred to the abomasum where first digestion occurs (138). Chigerwe et al. (97) calculated the optimal volume fed by esophageal tube for adequate transfer of passive immunity. Calves fed by esophageal tube require at least 150–200 grams colostral IgG, which can be translated to the recommendation of 3 L colostrum as soon as possible after birth, given the colostral Ig concentration is at least 50 mg/ml (97).

## Discussion

We attempted to provide a complete overview of all existing literature about on-farm colostrum management strategies and their effects on colostrum quality and calf immune status. With respect to milking methods, most studies included are observational population studies (26 out of 28). Results obtained from this type of study designs are often a valid reflection of field conditions and therefore have high external validity. The majority of the studies agrees that increasing the interval between calving and first milking decreases the concentration of IgG in colostrum. Probably, this reduction in IgG concentration is largely, but maybe not only, the result of a dilution effect. Dilution also explains decreased IgG concentrations in subsequent milkings. Therefore, milking the cow completely and as soon as possible after parturition is highly encouraged and will likely result in the highest colostral IgG concentrations. We discourage to measure colostral IgG quality by measuring only the first streaks, for it may not represent the quality of the complete milking of colostrum.

Regarding storage and treatments of colostrum after milking but before feeding it to the newborn, study designs in our results include mostly animal studies (*n* = 24) and laboratory studies (*n* = 26). Laboratory studies have high internal validity, and combined with evidence from animal studies and some population studies, we think the associations described here are quite strong. The most studied treatment of colostrum is the use of pasteurization. Effects of both duration and temperature have been studied extensively, of which the latter seems to affect colostral proteins the most. From the cited studies, sixteen studies looked into the effects of pasteurizing at 60°C on IgG levels, of which twelve concluded pasteurization can safely occur at 60°C without drastically affecting colostral IgG. Two out of four studies finding reduced colostral IgG concentrations looked into effects on calf serum IgG as well and concluded that calf serum IgG remained unaffected or even increased. Increasing temperature of pasteurization (>60°C) and prolonged duration of pasteurization lead to decreased levels of colostral and serum IgG. Pasteurizing small batches is associated with a smaller loss of immunoglobulins compared to pasteurizing larger batches, since larger batches require prolonged exposure to heat as opposed to smaller batches. From these results we conclude that heating up until 60°C results in a minimal reduction of colostral and calf serum IgG concentrations. Together with findings that total bacterial counts and coliform counts are diminished by heat treatment (47, 52), pasteurization could provide protection against neonatal disease development (46, 139, 140). Feeding pasteurized colostrum and milk for a prolonged period (21 days) results in long term health effects, including reduced morbidity, increased body weight and increased milk production during the first lactation (141).

Freezing does not, or only slightly affect colostrum IgG concentration or serum IgG levels. Thawing by au bain-marie method up to 40°C is best, using a microwave results in unevenly heated colostrum, leaving some parts to remain frozen and other parts to be heated to a degree that proteins denature. Similar to the pasteurization process, we advise to thaw (and thereby freeze) in small portions to reduce the duration of heat exposure. Repeated freeze/thawing is discouraged, as colostral IgG concentrations diminish after multiple freeze/thaw cycles. We did not find studies in which thawing at room temperature or in a refrigerator was studied, however it is known that total bacterial counts will increase over time when left at room temperature (38, 39). Effects of freeze/thawing on other colostral immune components are not well-understood, but seems to reduce immunity cells viability. A minority of studies involved the treatment of colostrum by fermentation, freeze drying or gamma radiation. Fermentation is a method not commonly applied, however freeze drying and gamma radiation are performed by commercial suppliers of fresh colostrum replacements. In this review we aimed to address colostrum practices with regard to fresh colostrum in on-farm situations, therefore these methods of colostrum treatment were not included within this research.

For administration procedures, we found 36 animal studies and 18 population studies. The animal studies were often randomized controlled studies and thus provide strong internal validation. Together with the population studies with high external validity, we considered this a substantial body of evidence. Most studies agree that the ability to absorb IgG is highest directly after birth and declines with time. The ability to transfer macromolecules such as immunoglobulins decreases due to the process of “gut closure” (8). With respect to suckling with the dam, when calves are not assisted they often lack vigor to start suckling in time and therefore are at risk of FPI. We recommend to use an active form of colostrum feeding either by nipple bottle or esophageal feeding, to ensure a sufficient volume is ingested. Both methods are suitable for obtaining adequate transfer of passive immunity, as out of the nine studies comparing these methods, the majority (6) reports no significant differences in IgG absorption by the calf. For normal healthy calves we advise to use a bottle for colostrum feeding; esophageal tube feeding can be an invasive procedure and should only be used when calves do not voluntarily ingest a sufficient amount of colostrum. Recommendations for feeding volume vary and each of these recommendations is based on a different principle, for example providing a certain mass of IgG or providing a volume adjusted to the size and birthweight of the calf. Following the first recommendation, the volume to be fed is determined according to the *quality* of the colostrum: when quality of colostrum equals or exceeds 50 g/L, a calf should ingest at least 2 or 3–4 L according to the recommendations to ingest 100 or 150–200 grams IgG in total, respectively. When colostrum quality is low (<50 g/L), even more should be fed divided over one or two extra feeding times. The second guideline depends on the *birthweight* of the calf. When the recommendation of feeding 8.5% of the BW is applied, 3.4 L is required for an average calf weighing 40 kg at birth. All of the guidelines described here provide an approximation of the volume to be administered. In fact, the volume that should be ingested to achieve serum IgG > 10 mg/mL, depends on a combination of many factors: the quality of the colostrum (105), the bodyweight (103), and the absorption efficiency of the calf, of which the latter is affected by the timing of feeding and possibly by volume (104) and bacterial content of the colostrum (62). A finite answer to the question how much colostrum should be given exactly, remains difficult. Because some of these determinants of calf serum IgG are hardly quantifiable in on-farm situations, such as the absorption capacity and related factors, it is even more important to at least measure those parameters that *can* be quantified, such as colostrum quality and the weight of the newborn calf. By doing so, a farmer can adjust the colostrum volume to satisfy requirements for each individual calf. Which method should be used to determine colostrum volume, whether calculated by birthweight or colostrum quality, is probably depending on the situation on farm, e.g., which parameters *can* be measured and/or adjusted by the farmer. Ideally, all parameters should be taken into account for determining individual colostrum intake.

Searching systematically for milking, storing and feeding strategies of colostrum enabled us to identify current gaps regarding knowledge on colostrum management. One of the limitations in this field of research, is that the emphasis lies on how concentration of immunoglobulin G in colostrum or eventually calf serum is influenced, while the effects on bioavailability and *in vivo* functionality are understudied. We know bacterial contamination of colostrum reduces intestinal uptake of immunoglobulins, however one of the functions of colostral immunoglobulins is to bind to pathogenic bacteria to prevent colonization. Hence, besides looking into systemic effects, local effects of colostrum immune components in the gastro-intestinal tract should be investigated as well. There is a growing body of literature that recognizes the transfer colostral leukocytes to newborns and its functionality. Liebler-Tenorio et al. found that colostral leukocytes are transferred through the epithelial barrier via follicle-associated epithelium of Peyer patches in the neonate gut (142). Protective effects of colostral leukocytes on neonatal immune development are described by Donovan et al. (77). In addition, Reber et al. proposed that presence of maternal leukocytes from colostrum was correlated with faster development of neonatal lymphocytes in the first week of life (143). Despite the promising effects of colostral leukocytes, very little is known about how colostrum management affects the functionality of these maternal cells in the newborn. We do know that uptake of colostral cells by the neonate is not limited by gut closure (144), however how storage methods or pasteurization affects viability or functionality of these maternal leukocytes remains uncertain. There are some limitations to our scoping review. Because of the magnitude of the available literature on colostrum management, it was impossible to cover *all* aspects related to colostrum management procedures and we had to delineate our scope. Therefore, we decided not to include, for example, the effects of treatment and storing on colostral microbiome or specific antibodies studied *in vitro*. Furthermore, in many systematic reviews, two independent persons carry out the search and selection process, we only utilized a single person. As we did not aim to quantify the combined search results in a meta-analysis procedure, we feel one person was sufficient for this scoping review.

This review was set out to evaluate current knowledge on-farm colostrum management processes and how these affect immune properties of colostrum. The findings of this study suggest that optimal colostral IgG can be achieved by milking colostrum directly after calving. If possible, we advise pasteurizing fresh colostrum for 30 min at 60°C. Colostrum can safely be stored by freezing and thawing by au bain-marie is recommended to minimize IgG loss. In on-farm situations, determining the volume that should be fed is difficult. The variables contributing to absorption of IgG by the newborn calf are many and include the quality of the colostrum, the bacterial contamination, the time interval between birth and first moment of feeding and the weight of the calf. Despite all knowledge regarding optimal colostrum management strategies, it remains difficult to predict effects of certain colostrum management choices in practice. We therefore recommend measuring the colostral quality and/or weighing the newborn calf and adjust the volume of feeding accordingly to ensure optimal colostrum intake for each individual calf. The main emphasis in this field of study is still on the concentrations of IgG, however consequences of colostrum management on colostral leukocytes and other colostral immune compounds are understudied.

## Author Contributions

LR designed and performed the systematic literature search and wrote the first manuscript. All authors contributed to the article and approved the submitted version.

## Conflict of Interest

The authors declare that the research was conducted in the absence of any commercial or financial relationships that could be construed as a potential conflict of interest.
