# Supplementary material for: A Scoping Review of On-Farm Colostrum Management Practices for Optimal Transfer of Immunity in Dairy Calves
Source: Front Vet Sci. 2021 Jul 19;8:668639. doi: 10.3389/fvets.2021.668639 (PMC8327206; doi:10.3389/fvets.2021.668639)
Supplement: Supplementary file 1 [file Table_1.docx]

Supplementary Material

1. **Supplementary tables**

## Supplementary table 1: Comparison of pasteurization methods and their effects on colostral immunoglobulin and serum immunoglobulin concentrations.

| Study | Pasteurizaion process | | | | | Calf feeding | Outcome | |
| --- | --- | --- | --- | --- | --- | --- | --- | --- |
|  | **Method** | **Comparison** | **Temperature** | **Time** | **Volume (L)** | **Volume** | **Colostral IgG** | **Serum IgG** |
| (Godden et al., 2003) | Batch pasteurizing system^^[[1]](#footnote-1)^^ | Fresh frozen colostrum | 63 | 30 | 57  95 | 2  4 | Reduced IgG  Larger effect for larger volumes pasteurized | Reduced IgG (2L feeding)  No effect (4L feeding) |
| (Bush et al., 1982) | Pasteurization | Fresh frozen colostrum | 63 | 30 | ? | ? | ? | Increased IgG |
| (Tyler et al., 2000) | Commercial Pasteurizer^^[[2]](#footnote-2)^^ | Fresh frozen colostrum | 63 | ? | 3 | 3 | NA | No effect |
|  | Commercial Pasteurizer^2^ | Fresh frozen colostrum | 76 | ? | 3 | 3 | NA | Reduced IgG |
| (McMartin et al., 2006) | Rapid Visco Analyser^[[3]](#footnote-3)^ | Fresh frozen colostrum | 59 | 120 | 0.05 | NA | No effect IgG | NA |
|  |  | Fresh frozen colostrum | 60 | 120 | 0.05 | NA | No effect IgG | NA |
|  |  | Fresh frozen colostrum | 61 | 120 | 0.05 | NA | No effect IgG | NA |
|  |  | Fresh frozen colostrum | 62 | 120 | 0.05 | NA | Loss of IgG | NA |
|  |  | Fresh frozen colostrum | 63 | 120 | 0.05 | NA | Loss of IgG | NA |
| (Johnson et al., 2007) | Batch pasteurizing system1 | Fresh frozen colostrum | 60 | 60 | 8 | 3.8 | No effect IgG | Increased IgG  Increased AEA  No effect IgM  No effect IgA |
| (Meylan et al., 1996) | Water bath | Fresh frozen colostrum | 63 | 30 | 0.05 | NA | Loss of IgG | NA |
| (Stabel, 2008) | Pasteurization | Fresh (suckling) | 65 | 30 | ? | 4 | NA | No effect IgG (1 month) |
| (Elizondo-Salazar and Heinrichs, 2009b) | Commercial batch pasteurizer^[[4]](#footnote-4)^ | Fresh frozen colostrum | 60 | 30 | 28 | 3.8 | No effect IgG_1_, IgG_2_ and total IgG | Increased IgG_1_, IgG_2_ and total IgG |
| (Elizondo-Salazar and Heinrichs, 2009a) | Commercial batch pasteurizer4 | Fresh frozen colostrum | 60 | 30 | 21 | 3.8 | No effect IgG_1_, IgG_2_ and total IgG | Increased IgG_1_, IgG_2_ and total IgG |
| (Elizondo-Salazar et al., 2010) | Water bath | Fresh frozen colostrum | 57 | 30  60  90 | 0.01 | NA | No effect IgG_1_ and IgG_2_  No effect IgG_1_ and IgG_2_  No effect IgG_1_ and IgG_2_ | NA |
|  | Water bath | Fresh frozen colostrum | 60 | 30  60  90 | 0.01 | MA | Reduced IgG_1_, not IgG_2_  Reduced IgG_1_, not IgG_2_  Reduced IgG_1_ and IgG_2_ | NA |
|  | Water bath | Fresh frozen colostrum | 63 | 30  60  90 | 0.01 | NA | Reduced IgG_1_ and IgG_2_ Reduced IgG_1_ and IgG_2_ Reduced IgG_1_ and IgG_2_ | NA |
| (Godden et al., 2012a) | Batch pasteurizing system1 | Fresh frozen colostrum | 60 | 60 | ? | NA | No effect | NA |
| (Godden et al., 2012b) | Batch pasteurizing system1 | Fresh frozen colostrum | 60 | 60 | ? | 3.8 | No effect | Increased IgG |
| (El-Zahar et al., 2014) | ? | Fresh colostrum | 63 | 30 | ? | NA | Reduced IgG | NA |
|  | ? | Fresh colostrum | 72 | 0.25 | ? | NA | Reduced IgG | NA |
|  | ? | Fresh colostrum | 100 | 10 | ? | NA | Reduced IgG | NA |
| (Gelsinger et al., 2014) | Commercial batch pasteurizer4 | Fresh colostrum | 60 | 30 | ? | 3.8 | No effect | Increased IgG_1_, IgG_2_ and total IgG  Increased AEA |
| (Gelsinger and Heinrichs, 2017) | Commercial batch pasteurizer4 | Fresh frozen colostrum | 60 | 60 | ? | 8% BW | Reduced IgG | No effect IgG  No effect AEA |
| (Sotudeh et al., 2018) | Water bath | Fresh colostrum | 60  55 | 30  60 | 0.25 | NA | Reduced IgG  Reduced IgG | NA |
| (Saldana et al., 2019) | Commercial batch pasteurizer4 | Fresh frozen colostrum | 60 | 30  60 | ? | 3.8 | Reduced IgG | Increased AEA |
| (Kryzer et al., 2015) | Perfect Udder system^[[5]](#footnote-5)^ | Fresh frozen or refrigerated colostrum | 60 | 60 | 3.8 | 3.8 | No effect | Increased IgG  Increased AEA |
|  | Batch pasteurizing system1 | Fresh frozen or refrigerated colostrum | 60 | 60 | 12.6 | 3.8 | No effect | Increased IgG  Increased AEA |
| (Donahue et al., 2012) | Batch pasteurizing system1 | Fresh refrigerated colostrum | 60 | 60 | ? | NA | No effect | NA |
| (Rafiei et al., 2019) | On farm colostrum pasteurization system^[[6]](#footnote-6)^ | Fresh frozen colostrum | 60 | 30 | 1.5 | 10% BW | No effect | Increased IgG  Increased AEA |
| (Mann et al., 2020a;Mann et al., 2020b) | Batch pasteurizing system1 | Fresh frozen colostrum | 60 | 60 | 4 | 8.5% BW | No effect | No effect IgG  No effect IgA |
| (Elsohaby et al., 2018) | Water bath | Fresh frozen colostrum | 60  63 | 30  60  30  60 | 0.01 | NA | No effect  No effect  Reduced IgG  Reduced IgG | NA |

| Study | Temperature in °C | Duration in minutes | LF Control Colostrum (mg/mL) | LF Heat Treated Colostrum (mg/mL) | LF serum Control colostrum (mg/mL) | LF serum Heat Treated colostrum (mg/mL) |
| --- | --- | --- | --- | --- | --- | --- |
| (El-Fattah et al., 2014) | 60 | 60 | 1.0 ± 0.1 | 0.67 ± 0.0 |  |  |
| (El-Fattah et al., 2014) | 63 | 30 | 1.0 ± 0.1 | 0.57 ± 0.0 |  |  |
| (Teixeira et al., 2013) | 63 | 60 | 0.28 | 0.21 |  |  |
| (El-Fattah et al., 2014) | 72 | 0.25 | 1.0 ± 0.1 | 0.13 ± 0.0 |  |  |
| (Lakritz et al., 2000) | 76 | 15 |  |  | 1.97 ± 1.0 | 0.42 ± 0.32 |

## Supplementary table 2: Effects of pasteurization on lactoferrin (LF) concentrations in colostrum and calf serum.

## Supplementary table 3: Comparison of freeze/thawing methods and their effects on colostral and/or serum IgG concentration.

| Study | Treatment | Comparison | Watt | Temp. start (°C) | Temperature reached (°C) | Time (min) | Volume (L) | Volume given to calf (L) | Effect Ig | Effect serum Ig |
| --- | --- | --- | --- | --- | --- | --- | --- | --- | --- | --- |
| (Jones et al., 1987) | Microwave | Microwave, Bain-marie | 650 | -20 | ? | 10 | 1 | NA | No effect IgG, IgM, lower IgA | NA |
|  | Microwave | Microwave, Bain-marie | 325 | -20 | ? | 17 | 1 | NA | No effect IgG, IgM, lower IgA | NA |
|  | Bain-marie | Microwave |  | -20 | 45 | 25 | 1 | NA | No effect IgG, IgM, higher IgA | NA |
| (Olson, 1989) | Bain-marie | Microwave | NA | -29 | 41 | 20 | 1.89 | 3.78 | No effect IgG1, IgA and IgM.  Lower IgG2 | Lower IgG1 and IgG2 (24h)  No effect at 48h |
|  | Microwave | Bain-marie | 312 | -29 | 41 | 45 | 1.89 | 3.78 | No effect IgG1, IgA and IgM.  Higher IgG2 | Higher IgG1 and IgG2 (24h)  No effect at 48h |
| (Haines et al., 1992) | Bain-marie | Fresh | NA | -20 | 37 | 30 | 0.02 | NA | No effect IgG, IgA and IgM | NA |
| (Holloway et al., 2001) | Bain-marie | Fresh | NA | -20 | 25 | ? | 1 | 4 | ? | No effect IgG (48h) |
|  | Bain-marie | Fresh | NA | -80 | 50, 37 | ? | ? | 1.89 | ? | No effect IgG |
| (Wiking and Pedersen, 2009) | Microwave | Unknown | 850 | 4°C | 40-42 |  | 4 | NA | No effect IgG | NA |
| (Balthazar et al., 2015) | Microwave | Fresh | 200 | -20 | 39 | 14,7 | ? | NA | 20% IgG1 loss | NA |
|  | Microwave | Fresh | 350 | -20 | 39 | 26,6 | ? | NA | 31% IgG1 loss | NA |
|  | Bain-marie | Fresh | NA | -20 | 40 | 65 | ? | NA | 8% IgG1 loss | NA |
|  | Bain-marie | Fresh | NA | -20 | 50 | ? | ? | NA | 14% IgG1 loss | NA |
|  | Bain-marie | Fresh | NA | -20 | 60 | ? | ? | NA | 15% IgG1 loss | NA |
|  | Bain-marie | Fresh | NA | -20 | 70 | 37 | ? | NA | 26% IgG1 loss | NA |

## Supplementary table 4: Comparison between esophageal feeding and other methods of feeding.

| Study | Year | N | Treatment | Volume given to calf within 12h (L) | Serum IgG/FPT | Better? |
| --- | --- | --- | --- | --- | --- | --- |
| (Logan et al., 1981) | 1981 | 31 | Esophageal feeder | 0,5-1 | Lower FPT in bottle and esophageal fed calves | Esophageal and bottle |
|  |  | 47 | Nipple bottle | 0,5-1 |  |  |
|  |  | 250 | Suckling | NA |  |  |
| (Bradley and Niilo, 1985) | 1985 | 31 | Esophageal feeder | 1 | No sig. difference within 48h | Neither |
|  |  | 31 | Suckling | NA |  |  |
| (Adams et al., 1985) | 1985 | 52 | Esophageal feeder | 10% BW | No sig. difference | Neither |
|  |  |  | Nipple bottle |  |  |  |
| (Besser et al., 1991) | 1991 | 334 | Esophageal feeder | 2,84 | 10,8% FPT | Esophageal feeder |
|  |  | 83 | Nipple bottle | 3,8 | 19,3% FPT |  |
|  |  | 165 | Suckling | NA | 61,4% FPT |  |
| (Kaske et al., 2005) | 2005 | 15 | Esophageal feeder | 4 | Higher IgG esophageal feeder | Esophageal feeder |
|  |  | 21 | Nipple bottle | 2 |  |  |
| (McGee et al., 2006) | 2006 | 83 | Esophageal feeder | 50 ml/kg | No sig. difference | Neither |
|  |  |  | Suckling | NA |  |  |
| (Elizondo-Salazar et al., 2011) | 2011 | 40 | Esophageal feeder | 3,8 | No sig. difference | Neither |
|  |  |  | Nipple bottle | 3,8 |  |  |
| (Chigerwe et al., 2012) | 2012 | 13 | Esophageal feeder | 2,2 | No sig. difference | Neither |
|  |  | 13 | Nipple bottle | 2,2 |  |  |
| (Bonk et al., 2016) | 2016 | 37 | Esophageal feeder | 3,5 | No sig. difference | Neither |
|  |  |  | Nipple bottle | 3,5 |  |  |

| (Desjardins-Morrissette et al., 2018) | 2018 | 10 | Esophageal feeder | 3 | No sig. difference | Neither |
| --- | --- | --- | --- | --- | --- | --- |
|  |  | 10 | Nipple bottle | 3 |  |  |
| (Shah et al., 2019) | 2019 | 5 | Esophageal feeder | 10% BW | Higher IgG esophageal feeder compared to suckling.  No sig. difference bottle vs esophageal feeder | Suckling< bottle < esophageal feeder |
|  |  | 5 | Nipple bottle | 10% BW |  |  |
|  |  | 5 | Suckling | NA |  |  |
| (Godden et al., 2009) | 2009 | 97 | Esophageal feeder | 1,5 | 1.5L: Higher IgG for bottle feeding. 3L: No sig. difference in feeding methods | Depends on the volume given |
|  |  |  | Esophageal feeder | 3 |  |  |
|  |  |  | Nipple bottle | 1,5 |  |  |
|  |  |  | Nipple bottle | 3 |  |  |

1. Batch pasteurizing system, DT Silver, DairyTech Inc., Windsor, CO [↑](#footnote-ref-1)
2. Pres-Vac Home Pasteurizer, Schlueter Company, Janesville, WI 53545, USA [↑](#footnote-ref-2)
3. Rapid Visco Analyser, Newport Scientific, Warriewood, Australia [↑](#footnote-ref-3)
4. Girton Manufacturing Co., Millville, PA [↑](#footnote-ref-4)
5. The Perfect Udder colostrum management system, developed by Dairy Tech Inc. (Greeley, CO) [↑](#footnote-ref-5)
6. On farm pasteurization system (V4, Shirmark Group) [↑](#footnote-ref-6)
